# Editorial: Surgical oncology in the elderly: the state of the art and future challenges

**DOI:** 10.3389/fonc.2023.1224278

**Published:** 2023-06-13

**Authors:** Cosimo Sperti, Lucia Moletta, Felix Berlth

**Affiliations:** ^1^ Department of Surgery, Oncology and Gastroenterology, Hepato-Bilio-Pancreatic (HPB) Unit, University of Padua, Padova, Italy; ^2^ Department of Surgery, Oncology and Gastroenterology, 1^st^ Surgical Clinic, University of Padua, Padova, Italy; ^3^ Mainz University, Johannes Gutenberg Universitat Mainz, Mainz, Germany

**Keywords:** surgical oncology, elderly, esophageal cancer, gastric cancer, pancreatic cancer, colorectal cancer

The number of old patients with cancer is increasing since population ageing is a common event throughout the world. It is estimated that the number of persons aged 80 years or over increase more than threefold between 2017 and 2050, rising from 137 million to 425 million (1). So, more elderly patients with cancer requiring a surgical evaluation are expected in clinical practice. However, surgical oncology in the elderly presents several problems. Undoubtedly, the decline in physiological systems and the presence of comorbidities have an impact in the surgical management of cancer patients together with the tolerance to oncological treatments (2). Moreover, we are lacking clear evidence-based informations about this topic because only small subset of geriatric cancer patients has been included into clinical trials (3).

The surgical management of elderly patients with cancer is still a challenge and frequently troubles the surgeon. The current problem is the preoperative assessment of elderly patients and the search for prognostic factors suggestive for frailty or other factors influencing perioperative outcome (4). The role of age as prognostic index in old patients undergoing surgical treatment shows contrasting results. Some studies reported age as an independent prognostic factor for morbidity and mortality after surgery, while other Authors have suggested that elderly patients with healthy conditions are candidate for surgical resection with the same surgical risk of younger patients (5, 6).

Another problem is the feasibility of multimodality therapy (neoadjuvant or adjuvant therapy) in such elderly patients. Toxicity of chemotherapy and/or radiotherapy regimens may be increased in geriatric patients and limited physiological reserve make difficult to complete oncological treatments (7). Finally, the role of minimally invasive surgical resections in the elderly remains to be assessed (8).

Within this Research Topic, the manuscript by Hu et al. reports a successful laparoscopic resection of two malignancies (left hepatectomy and total hysterectomy) in a 75-year-old woman with intrahepatic cholangiocarcinoma and endometrial cancer, emphasizing that laparoscopic complex operation may be offered to elderly, well-selected patients.

The work of Jin et al. focused on the impact of malnutrition on outcome after major surgical procedures for cancer. The Authors showed that Protein-Energy Malnutrition (PEM) was associated with increased risk of mortality, major complications, higher total cost, and longer length of stay.

The paper by Zhang et al. analyzed the local treatment of ductal carcinoma *in situ* (DCIS), including breast surgery, axillary lymph node (ALN) surgery, and radiotherapy in different subgroups of aged patients. Associations with clinicopathological findings and outcome after surgery were evaluated. Age and tumor size were independent factors influencing the breast and ALN surgery. Compared with patients aging 60-69, octogenarian patients underwent more breast conserving surgery (BCS) and less ALN surgery. Age was the only factor associated with the radiotherapy decision after BCS in elderly patients with DCIS.

Gallina et al. reported their experience of robotic pulmonary lobectomy in a population of 103 patients older than 75 years. Thirty-five patients reported postoperative complications without mortality. The factors that could predict the complication rate were the predicted postoperative FEV1 and the nodal disease. The Authors outlined that the predicted postoperative FEV1 and the preoperative staging should be carefully evaluated in order to improve postoperative outcome.

The value of minimally invasive surgery in elderly patients has been reported by Capovilla et al. In two high-volume centers from Italy and Germany, 160 patients older than 75 years underwent open (n=102), laparoscopic (MIE; n=249) or robotic (RAMIE; n=34) esophagectomy. Among elderly patients MIE/RAMIE were significantly associated with a lower overall morbidity, less pulmonary complications and a shorter hospital stay.

Xu et al. evaluated the effect of surgery for gallbladder cancer in elderly patients (> 70 years). Patients with surgery had significantly longer overall survival (OS) and cancer-specific survival (CSS) than those without surgery, especially patients aged 70-84 years old. An age >85 years was significantly associated with poor OS and CSS.

The morbidity and mortality after gastrectomy for gastric or distal esophageal cancer in patients aged >75 years in Germany, are presented in the work of Berlet et al. In a total of 67389 gastrectomized patients, the rate of patients aged 75 years or older was 51.4%. The hospital mortality of elderly patients was significantly higher, as well as the general complications and the need for resuscitation. Systematic D2 dissection, peritonectomy and hyperthermic intraperitoneal chemotherapy were less frequently performed in older patients compared to the younger countpart.

The paper by Bao et al. evaluated 144 patients who underwent surgical resection following nCRT for mid-low rectal cancer. The correlations between BMI and radiologic fat parameters with pathologic response and survival were investigated, without showing any difference in terms of OS and disease -free survival. Age did not correlate with pathologic response or survival.

Correlation between age and postoperative outcome in colorectal cancer patients was investigated by (Turri et al.) In a large cohort of 1482 patients operated for colorectal cancer, postoperative mortality was low in octogenarians (3.2%). OS decreased with advancing age. Although the results of surgery in elderly patients were acceptable, OS is strongly dependent on age. Compared to younger patients, mortality in older people was frequently due to competing causes rather than to cancer-related managements.

In order to better identify the prognostic factors in elderly operated for colorectal cancer, Mao and Lan performed a systematic review and meta-analysis on the prognostic value of the Geriatric Nutritional Risk Index (GNRI). The study included ten reports for a total of 3802 patients. Meta-analysis showed a significant poor overall survival (and disease-free survival) and higher risk of complications in patients with low GNR1. When a subgroup analysis based on age was performed, the results did not change.

In conclusion, all contributions to this Research Topic suggest that major cancer surgery may be safe and feasible in elderly patients, but the risk of postoperative complications undoubtedly exists and worries surgeons and oncologists. Despite oncological outcome appears not influenced by patients’ age, we have few informations about the quality of life of these patients. Future studies are needed to identify simple indicators to stratify patients for more precise surgical risk and formulate personalized treatment plans.

## Author contributions

All authors listed have made a substantial, direct, and intellectual contribution to the work and approved it for publication.
